# In glaucoma patients, low blood pressure is accompanied by vascular dysregulation

**DOI:** 10.1007/s13167-018-0155-5

**Published:** 2018-11-12

**Authors:** Tatjana Binggeli, Andreas Schoetzau, Katarzyna Konieczka

**Affiliations:** Department of Ophthalmology, University of Basel, Mittlere Strasse 91, CH-4031 Basel, Switzerland

**Keywords:** Glaucoma, Normal tension glaucoma, Nailfold capillaroscopy, Blood pressure, Vascular dysregulation, Flammer syndrome, Predictive diagnostics, Predictive preventive personalized medicine

## Abstract

**Background:**

There are many risk factors contributing to glaucomatous optic neuropathy. Beside increased intraocular pressure, vascular factors play a prominent role, particularly low blood pressure (BP), and vascular dysregulation. Both of them are essential components of the Flammer syndrome. The aim of this retrospective study was to evaluate whether in glaucoma patients there is a relationship between vascular dysregulation and the BP.

**Methods:**

Medical records of 57 unselected glaucoma patients were retrospectively studied.

**Results:**

Based on the outcome of the capillaroscopy, patients were divided in a group of patients with vascular dysregulation, also called long-stoppers (flow cessation for 13 s or more), and a group of patients with normal vascular regulation, also called short-stoppers (flow cessation for 12 s or less). BP was significantly lower in the group of long-stoppers than in the group of short-stoppers. This applies for both systolic (*p* = 0.028) and diastolic BP (*p* = 0.036). The regression analysis revealed also a significant inverse relationship between the duration of blood flow cessation and the systolic (*p* = 0.025) and diastolic BP (*p* = 0.016). After adjustment for age, gender, use of antihypertensive therapy, and excluding patients taking calcium channel blockers, the relationship was still significant for systolic (*p* = 0.025) and diastolic BP (*p* = 0.003).

**Conclusions:**

In glaucoma patients, vascular dysregulation (as defined by response in the nailfold capillaroscopy to a cold provocation) and low BP are statistically related. This is in line with the observation that Flammer syndrome subjects have both primary vascular dysregulation and low BP and that Flammer syndrome is a risk factor for glaucomatous optic neuropathy, at least in normal tension glaucoma patients. The detection of vascular factors in glaucoma patients may lead to a more efficient treatment, better tailored to the individual patient.

## Introduction

There is growing evidence that besides intraocular pressure (IOP), vascular factors are also involved in the pathogenesis of glaucomatous optic neuropathy (GON) [1]. Two vascular factors have been a major focus of study: low blood pressure (BP) [2] and primary vascular dysregulation [3]. Both factors are essential components of Flammer syndrome [4, 5], and the prevalence of Flammer syndrome is higher in glaucoma patients, particularly in patients with normal tension glaucoma (NTG) [6]. Although these two factors independently contribute to GON [7], a pilot study suggested that the prevalence of the two factors might be related [8].

Primary vascular dysregulation mainly involves the microcirculation [5]; therefore, nailfold capillaroscopy is a preferred method for diagnosis. Reduced blood flow velocity in the nailfold capillaries of glaucoma patients, particularly of glaucoma patients with NTG, was described decades ago [9] and was recently confirmed in a large-scale study [10]. Local cooling is one of the trigger factors most often used in combination with capillaroscopy [11] to diagnose different varieties of vascular dysregulation, including primary vascular dysregulation [5]. Prolonged stoppage of blood flow in the nailfold capillaries after cooling has been described in glaucoma patients, particularly in NTG patients [9].

These vascular factors are becoming increasingly important as the proportion of NTG in glaucoma increases. A recent update on the prevalence, etiology, diagnosis, and monitoring of NTG revealed that the proportion of NTG varies between countries from 30 to 90% [12].

The goal of this retrospective study was to test the relationship between vascular dysregulation and BP [3, 5, 13] in glaucoma patients. For this reason, we analyzed the duration of blood flow cessation in nailfold capillaries induced by local cooling and systolic and diastolic BP values.

## Methods

### Study participants

We retrospectively studied the medical records of 57 glaucoma patients (35 women and 22 men) referred to the Department of Ophthalmology at University Hospital Basel, Switzerland, and aged between 17 and 92 years. Glaucoma was diagnosed if typical glaucomatous excavation of the optic nerve head and corresponding visual field defects were present. Each patient had a documented comprehensive ophthalmologic evaluation with the following examinations: slit lamp, funduscopy, best corrected visual acuity, and IOP, as well as a nailfold capillaroscopy and BP measurement.

Ethical approval for the study project was obtained from the local medical ethics committee “Ethikkommission Nordwest- und Zentralschweiz/EKNZ” (EKNZ: BASEC 2016-00531). The study was designed and conducted in accordance with the tenets of the Declaration of Helsinki.

### Nailfold capillaroscopy

The nailfold capillaries were studied using a light microscope (Ernst Leitz Wetzlar Type 307-143-003, Ernst Leitz GmbH, Germany) attached to a television monitor, which was coupled to a video recorder. The television pictures depicting blood flow in the capillaries were videotaped and analyzed afterwards. The examinations were performed in a room with a constant temperature of 23 °C. Before the examination, the patients were acclimatized in this room for 30 min.

**Fig. 1:**
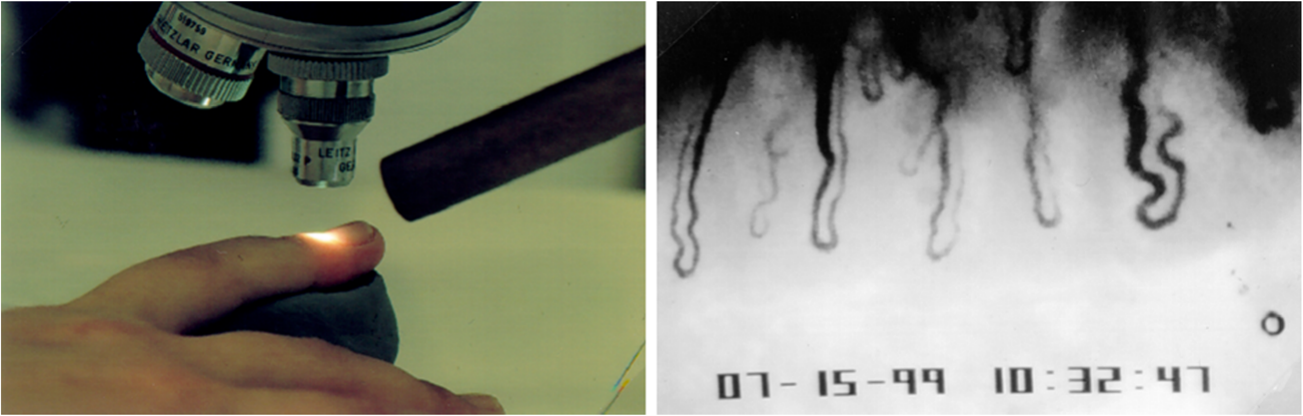
Nailfold capillaroscopy. Left, the capillaries are studied using a light microscope. For the cold provocation, decompressed air from a tube is blown over the nailfold. Right, picture of nailfold capillaries taken from the video (from [14])

The skin of the nailfold was made transparent using a drop of oil. The blood flow in the capillaries running parallel to the skin surface was observed. After the baseline blood flow recording, the nailfold area was cooled for 60 s by blowing decompressed carbon dioxide of approximately − 15 °C over the nailfold [9] (Fig. 1).

For clinical purposes, blood flow standstill between 0 and 12 s was considered normal [11], and therefore the exact time was not recorded in the chart. For this retrospective study however, 5 s (which represents about the mean stop-time in this group) was set arbitrarily as stop-time, and these patients were termed short-stoppers (clinically, this means they do not have vascular dysregulation [5]). A blood flow standstill of 13 s or longer was clinically considered pathological and therefore exactly recorded, allowing us to use these stop-times for analysis, and these patients were termed long-stoppers (clinically, this means they have vascular dysregulation). The mean of the stop-times of the individual capillaries in the nailfold was termed mean stop-time (mST) of the patient.

### Blood pressure measurements

BP was measured directly before capillaroscopy using an Omron blood pressure monitor (Kyōto, Japan).

### Statistical methods

Descriptive statistics are presented as counts and frequencies for categorical data and medians [min, max] for metric variables. Overall *p* values correspond to the Kruskall-Wallis test (for median) and chi-squared test. In order to predict systolic or diastolic BP from mST or “stop-time group”, linear mixed-effects models were performed. mST was log-transformed to achieve more symmetric distribution. Mixed-effects models are suitable for analyzing repeated measurement data (here data from the left and right eyes). Results were adjusted for age, gender, use of antihypertensive therapy, and excluding patients under calcium channel blockers. Results are presented as mean differences between “stop-time groups” with 95% confidence intervals and *p* values. Using log (mST) as a predictor, only *p* values were reported. A *p* value < 0.05 was considered significant. All evaluations were conducted using the statistical software R version 3.1.1 [15].

## Results

Among our glaucoma patients, 34 (60%) were short-stoppers and 23 (40%) were long-stoppers. Among the short-stoppers were 21 women (61.8%) and 13 men (38.2%). Among the long-stoppers were 14 women (60.9%) and 9 men (39.1%). The median IOP was 13.0 [min = 10.0; max = 18.5] mmHg in long-stoppers and 13.5 [10.0; 23.5] mmHg in short-stoppers; the difference was not statistically significant (*p* = 0.57). Glaucoma patients taking antihypertensive treatment had a significantly higher systolic BP (*p* = 0.015, mean difference = 8.29, 1.66 to 14.92) despite treatment (differences in diastolic BP were not significant).

**Fig. 2:**
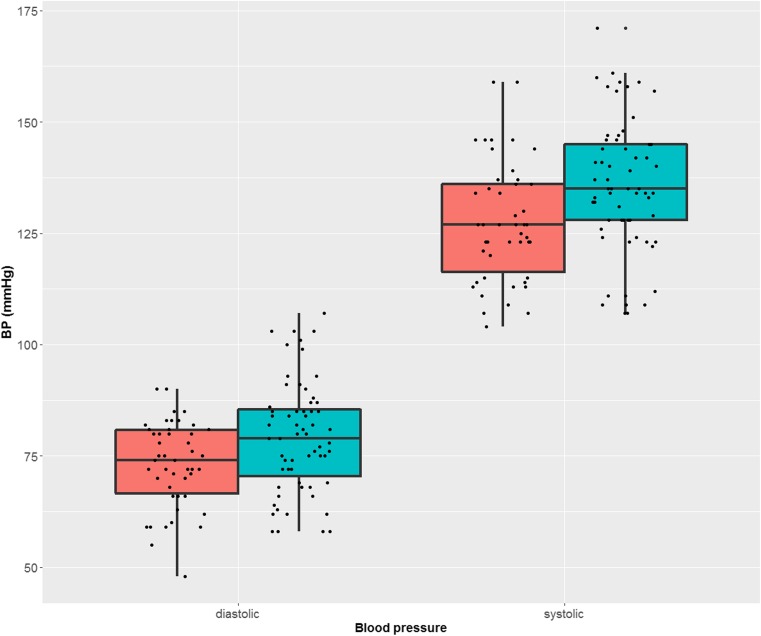
Diastolic (left) and systolic (right) blood pressure. In red is the blood pressure of glaucoma patients with prolonged blood flow cessation in the nailfold capillaries and in green the blood pressure of glaucoma patients with physiological response of the capillaries to cold. In other words, glaucoma patients with vascular dysregulation had on the average a significant lower blood pressure. The results are presented as boxplots. BP blood pressure

BP was significantly lower in long-stoppers than in short-stoppers. This applies to both systolic (*p* = 0.028, mean difference 8.47, CI 0.96 to 15.97) and diastolic BP (*p* = 0.036, mean difference 6.45, CI 0.42 to 12.48). The results are depicted in Fig. 2. The regression analysis also revealed a significant inverse relationship between the duration of blood flow cessation in capillaroscopy and BP: systolic (*p* = 0.025) and diastolic (*p* = 0.016). The longer the standstill in the nailfold capillaries, the lower the systolic and diastolic BP. After adjustment for age, gender, use of antihypertensive therapy, and excluding patients taking calcium channel blockers, the relationship was still significant for systolic (*p* = 0.025) and diastolic BP (*p* = 0.003).

## Discussion

This retrospective study with a group of unselected glaucoma patients reveals that patients with vascular dysregulation have on average lower systolic and diastolic BP. Vascular dysregulation was diagnosed in this study with the help of nailfold capillaroscopy combined with a local cold provocation. Dysregulation was assumed if the blood in capillaries stopped for more than 12 s during or after cooling. In addition, the regression analysis revealed a significant inverse relationship between the duration of blood flow cessation and BP. This relationship remained statistically significant after adjustment for age, gender, use of antihypertensive therapy, and excluding patients taking calcium channel blockers. Patients with calcium channel blockers were excluded, because calcium channel blockers have a major impact on the outcome of capillaroscopy [16].

Primary vascular dysregulation and low BP [5, 13] are two core elements of Flammer syndrome [4, 17], which has been described as linked to NTG [6]. The results of this study support the assumption that the prevalences of these two signs are interrelated.

Lower blood flow velocity in the nailfold capillaries of glaucoma patients was already described decades ago [9, 18, 19]. This has recently been confirmed by a multicenter study [10]. Lower blood flow velocity in glaucoma patients, particularly in patients progressing despite a normal IOP, has also been observed in other vascular beds, such as the retroocular vessels [20]. To diagnose vascular dysregulation however, blood flow regulation must be challenged. This has often been done by a cold provocation [9, 11]. Longer blood flow cessation after cooling in glaucoma patients, particularly in patients with NTG, has been observed for a long time [9].

The relationship between BP and GON has also been studied [2, 21–25]. Although arterial hypertension, like other classical vascular risk factors (such as dyslipidemia and diabetes mellitus), is associated with elevated IOP [21, 22], it is rather low BP that represents a direct risk for GON. Low BP and low perfusion pressure are well-studied risk factors for the occurrence and progression of GON [2, 23–28]. Increased blood flow fluctuation, for example due to over-dipping at night, is particularly relevant to GON [23, 28] as this leads to instable oxygen supply increasing oxidative stress [29]. In this study, however, we measured BP only once, before the capillaroscopy with a cold provocation was done.

Although primary vascular dysregulation and low BP are risk factors that contribute independently to GON [7], the prevalence of these factors is interrelated, an observation confirmed in this study. Patients with Flammer syndrome not only have lower BP on average [30] but also higher retinal venous pressure [31], further reducing perfusion pressure. Unfortunately, patients included in this study had no measurements of retinal venous pressure. Subjects with Flammer syndrome also more often have optic nerve compartment syndrome [32], and they respond well to low doses of calcium channel blockers [32, 33]. Future prospective studies should take all these relevant parameters into account in order to see the relationships among them and GON.

In glaucoma, especially in NTG patients with Flammer syndrome as well as in glaucoma patients progressing despite a normalized IOP, measuring of BP and quantification of regulation of blood flow are important diagnostics. Both factors are in themselves and even more in their combination risk indicators for the development of future glaucoma losses. This makes them important therapeutic targets and enables individual, personalized treatment.

## Conclusion

In glaucoma patients, vascular dysregulation (as defined by nailfold capillaroscopy response to cold provocation) and low BP are statistically related. This is in line with the observation that subjects with Flammer syndrome have both primary vascular dysregulation and low BP and that Flammer syndrome is a risk factor for GON, at least in NTG patients. Examining vascular aspects in glaucoma patients, such as BP and quantification of regulation of blood flow, may lead to a more efficient treatment tailored to the patient and prevent progression of glaucoma damage, especially in NTG patients. Targeted predictive vascular diagnostics may lead to better recognition of patients with increased risk. This may ultimately lead to a more personalized treatment.

## Abbreviations

*BP*: blood pressure
*GON*: glaucomatous optic neuropathy
*HTG*: high tension glaucoma
*IOP*: intraocular pressure
*NTG*: normal tension glaucoma
